# Pharmacokinetics of esomeprazole in goats (*Capra aegagrus hircus*) after intravenous and subcutaneous administration

**DOI:** 10.3389/fvets.2022.968973

**Published:** 2022-12-15

**Authors:** Rachel Fladung, Joe S. Smith, Melissa T. Hines, Windy Michelle Soto-Gonzalez, Bryanna Fayne, Rebecca R. Rahn, Olivia Grace Escher, Lainey Harvill, Joan Bergman, Jessica D. Garcia, Amanda Jo Kreuder, Sherry Cox

**Affiliations:** ^1^Large Animal Clinical Sciences, College of Veterinary Medicine, University of Tennessee, Knoxville, Knoxville, TN, United States; ^2^Biomedical Sciences, College of Veterinary Medicine, Iowa State University, Ames, IA, United States; ^3^Biomedical and Diagnostic Sciences, College of Veterinary Medicine, University of Tennessee, Knoxville, Knoxville, TN, United States; ^4^Veterinary Microbiology and Preventive Medicine, College of Veterinary Medicine, Iowa State University, Ames, IA, United States

**Keywords:** proton pump inhibitor, ulcer, goat (*Capra aegagrus hircus*), pharmacokinetics, esomeprazole, ruminant

## Abstract

**Background:**

Stressed and hospitalized goats are at risk of developing abomasal (gastric) ulceration, but there is a paucity of pharmacokinetic studies for proton pump inhibiting drugs, such as, esomeprazole in goats.

**Objectives:**

The objectives for this study were to estimate plasma pharmacokinetic parameters for esomeprazole in adult goats after intravenous (IV) and subcutaneous (SQ) administration. A secondary objective was to describe the plasma kinetics of the metabolite esomeprazole sulfone after IV and SC administration in goats.

**Materials and methods:**

Esomeprazole was administered to 5 adult goats in a crossover study at doses of 1 mg/kg IV or 2 mg/kg SC. Plasma samples were collected over 36 h and analyzed via reverse phase HPLC to determine concentrations of esomeprazole and esomeprazole sulfone. Pharmacokinetic parameters were derived via non-compartmental analysis.

**Results:**

Following IV administration, mean values for plasma clearance (Cl), elimination half-life [T1/2 (λz)], C0, and volume of distribution (*V*_*z*_) of esomeprazole were estimated at 24.9 mL/min/kg, 6 min, 2.324 μg/mL, and 0.23 L/kg, respectively. After SC administration elimination half-life, maximum concentration (Cmax) and time to maximum concentration (Tmax) of esomeprazole were estimated at 29 min, 1.038 μg/mL, and 22 minutes respectively. Maximum concentrations of the sulfone metabolite were 32 and 18 ng/mL after IV and SC administration.

**Conclusion:**

Esomeprazole was rapidly eliminated from plasma after both IV and SC injection in goats. The elimination half-life in goats appears to be shorter than reported in dogs, as well as less than that reported for pantoprazole in goats. The sulfone metabolite was detected and also rapidly eliminated from the plasma after both IV and SC administration. Additional pharmacodynamic investigations are needed to determine the efficacy of esomeprazole on abomasal (gastric) acid suppression in goats and could include larger doses or additional routes of administration.

## Introduction

Abomasal (gastric) ulceration is a multifactorial disease of many ruminant species, as well as a common cause of morbidity and mortality. Prevalence of abomasal ulcers varies substantially because of differences in populations, but abomasal ulcers can be found in ruminants of all ages and production systems (1–4). Clinical signs range from mild (anorexia/hyporexia) to severe (acute death) but are often vague and difficult to interpret definitively as indicators of abomasal ulceration. Contributing to ulceration include factors such as age, weather, housing, stress, trauma, nutrition, bacterial overgrowth, and the administration of a non-steroidal anti-inflammatory drug (NSAID) (5–7). Small ruminants placed in a new environment, such as a hospital, can experience high levels of physiological stress, and many of these hospitalized patients also receive an NSAID as part of their treatment protocol. NSAIDs inhibit the synthesis of prostaglandins; prostaglandins within the abomasum act in multiple ways to protect the mucosal lining (1). A reduction in these protective mechanisms leaves the mucosal surface susceptible to damage by the acidic environment of the abomasum. Despite the risk of abomasal ulceration in these patients, there are no approved treatments for this condition in small ruminants.

Proton pump inhibitors (PPIs) such as pantoprazole and omeprazole are routinely used by large animal clinicians to treat gastric ulceration in horses and many ruminant species, including sheep, goats, camelids, and cattle (7–10). However, pharmacokinetic and pharmacodynamic studies of the effects of PPIs in small ruminants are limited. An investigation of pantoprazole administered to goats found plasma clearance of the drug to be lower for small ruminants than for members of other species, such as foals, calves, and alpacas (6). This study also reported that the elimination half-life was less than reported in other species, and that the concentration of intravenously or subcutaneously administered pantoprazole was undetectable after 4 h in all animals (6). A previous study examined the pharmacokinetic and pharmacodynamic properties of intravenously (IV) and subcutaneously (SC) administered pantoprazole in alpacas (8). In that study, pantoprazole, administered IV or SC, reached therapeutic concentrations in the plasma and significantly increased the pH of the third compartment. However, the availability of pantoprazole and esomeprazole can be limited, and other PPIs, such as esomeprazole remain uninvestigated in goats.

Pharmacokinetic studies of esomeprazole, the S-enantiomer of omeprazole, administered to human patients show that esomeprazole has a reduced first-pass hepatic metabolism, slower plasma clearance, and increased area under the curve (AUC) plasma concentration than that of omeprazole (11). Esomeprazole will accumulate in the acidic secretory canaliculi of the gastric parietal cells and binds irreversibly to H+/K+ ATPase enzymes (11). A study showed that esomeprazole has promising results in maintaining an elevated pH of gastric juice of horses (12). In that study, esomeprazole magnesium administered orally (60 or 80 mg) in horses maintained gastric pH of 5 or higher for up to 6 h post administration (12). No adverse reactions were found after administration of the drug. Esomeprazole was also demonstrated to raise gastric pH to 4 or above after being administered intravenously in horses (12).

Despite the potential use of esomeprazole in ruminants, currently no studies report the pharmacokinetics in goats. Esomeprazole is proposed for investigation as a therapeutic to utilize in hospitals to prevent the development or perpetuation of abomasal ulceration. Esomeprazole is investigated as a medication that may have prolonged therapeutic concentrations in goats than other proton pump inhibitor medications. Based on previous work with pantoprazole in alpacas and goats, as well as comparative dosing, a similar dose was proposed for pharmacokinetic determination in goats. No short-term side effects have been associated with the use of the proton pump inhibitor class of drugs in small ruminants such as goats, but more studies are necessary to confirm this observation. The primary objective of this study was to determine the plasma pharmacokinetics of esomeprazole in healthy adult goats after single intravenous (IV) and subcutaneous (SC) administration. An additional, secondary objective of this study was to investigate the pharmacokinetics of the sulfone metabolite after IV and SC administration of esomeprazole in healthy adult goats.

## Materials and methods

### Animals

The protocol was reviewed and approved by the Institutional Animal Care and Use Committee (IACUC) of the University of Tennessee, Knoxville (Protocol number: 2825-0221). Five healthy adult goats were utilized for this study. Three goats were pygmies and two were pygmy-crosses. The weights were 42.1 ± 6.1 kgs and ages of the goats were 3.2 ± 0.7 years. Two goats were castrated males and three of the goats were intact females. Goats were sourced from the teaching herd of goats maintained at the University of Tennessee's Veterinary Research and Education Center. Diet during the study consisted of *ad libitum* grass hay. None of the animals had been medicated within the four weeks prior to the study and all were current on vaccination for *Clostridium perfringens* types C and D, as well as tetanus.

### Drug administration and sampling

Before enrollment for the study, all goats were deemed healthy based on physical examination by a board-certified large animal veterinary specialist. Prior to intravenous administration, all goats had intravenous jugular catheters aseptically placed as previously reported, (13) with one catheter designated for blood collection and another designated for drug administration. Esomeprazole was reconstituted to a concentration of 8 mg/mL in the manufacturer's vial with 5 mL of 0.9% sodium chloride (Sodium Chloride Injection, USP). A 1 mg/kg dosage of esomeprazole (Esomeprazole Sodium, Mylan Int., USA) was administered intravenously to each goat. Blood samples were collected at 0, 5, 10, 20, 30, and 45 min and 1, 1.5, 2, 3, 4, 8, 12, 18, 24, and 36 h after drug administration. Blood samples were placed into lithium heparin tubes after collection, immediately placed on ice for several min and then immediately spun down and transferred to cryogenic vials for storage at −80° C until analysis. After a minimum 10-day washout period, goats were crossed over with a single intravenous catheter placed, and a 2 mg/kg dose of esomeprazole administered subcutaneously in the left axillary region caudal to the elbow. After administration, blood samples were collected and preserved as described for the intravenous administration.

### Sample analysis

Analysis of esomeprazole and its metabolite in plasma samples was conducted using reverse phase high performance liquid chromatography (HPLC) method based on a method previously validated for pantoprazole in goat plasma (14). The system consisted of a 2,695 separations module, and a 2,487 UV absorbance detector (Waters, Milford, MA, US). The compounds were separated on a Symmetry C_18_ (4.6 x 150 mm, 5 μm) column with a 5 μm Symmetry C_18_ guard column (Waters, Milford, MA, US). The mobile phase was a mixture of 20 mM ammonium acetate and acetonitrile (75:25). The flow rate was 1 mL/min and absorbance was measured at 290 nm.

Esomeprazole and its metabolite were extracted from plasma samples using a liquid-liquid extraction method. Samples that were previously frozen were thawed, vortex-mixed, and 100 μl of plasma was transferred to a 13 x 100 mm screw top tube followed by 10 μl of tinidazole (internal standard, 10 μg/mL) and 1 mL chloroform. The tubes were rocked for 10 min and then centrifuged for 10 min at 1,000 x g. The organic layer was transferred to a glass tube and evaporated to dryness with nitrogen gas. Samples were reconstituted in 225 μL of mobile phase and 100 μL was analyzed.

Method validation was performed according to the FDA Bioanalytical Guidelines (FDA) (15). Standard curves for the plasma analysis were prepared by fortifying untreated, pooled plasma with esomeprazole and its metabolite, which produced a linear concentration range of 5–5000 ng/mL. Recovery, accuracy and precision were determined by analyzing five replicates at a low, medium, and high concentrations within the concentration range of the curve. Recovery was calculated as the percentage of the drug response after extraction compared to the response of the drug in the standard at a known concentration. The quality control (QC) concentrations used were 15, 75, 300, 1,300 and 4,000 ng/mL. The recovery for esomeprazole ranged from 99% ± 2 to 101% ± 5 while the range for the metabolite was 99% ± 2 to 103% ± 5 for 15, 75, 300, 1,300 and 4,000 ng/mL, respectively. The average recovery for the internal standard was 99% ± 2. The accuracy of the assay was within 100, 99, 102, 102, and 101% for esomeprazole and 106, 105, 105, 103, and 103% for the metabolite for the QC concentrations used. The precision of the assay was (expressed as CV%) 0.77, 4.09, 2.62, 7.32, and 7.93% for esomeprazole and 5.99, 8.55, 2.47, 3.61, and 7.58% for the metabolite for the QC concentrations. The lower limit of quantification for both was 5 ng/mL.

### Pharmacokinetic analysis

The pharmacokinetic parameters for esomeprazole in goats were determined from plasma time vs. concentration data as previously described for non-compartmental analysis (6, 9, 16). Analysis of pharmacokinetics for each individual was performed with commercial modeling software using a statistical moments approach (PKanalix, Monolix Suite 2020R1, Lixoft, France).

Standard PK parameters were generated for individual animals, as previously reported (6, 9):

1. Maximum concentration extrapolated to time zero, C0 (esomeprazole).
2. Area under esomeprazole concentration–time curve, AUC_last_ and AUC_inf_.
3. Area under the moment curve, AUMC_inf_.
4. Esomeprazole mean residence time, MRT = AUMC_inf_/AUC_inf_.
5. Esomeprazole terminal half-life, T1/2 (λ*z*)) = ln (2)/λ*z*.
6. Esomeprazole systemic clearance, CL = Dose/AUC_inf_.
7. Also reported: Volume of distributionand Bioavailability (F), with Bioavailability calculated as, AUC_SC_/AUC_IV_ x Dose_IV_/Dose_SC_. A log trapezoidal rule was used for data analysis to estimate the area under the esomeprazole time-curves. Summary statistics on the individual PK parameters were performed thereafter to derive the geometric mean, median, and (min–max) range. Pharmacokinetic parameters for the sulfone metabolite, were determined from plasma time vs. concentration data as previously described (6, 17).

## Results

### Animal health

During the duration of the study no adverse health effects were observed in any of the goats. Of the animals included in the study, no animal displayed inappetence or abnormal attitude or mentation. There was no evidence of the development of edema, injection site reactions or catheter site reactions. There was no evidence of anaphylactic reaction or adverse reactions throughout this study.

### Pharmacokinetic parameters

Table 1 displays the geometric mean, median, minimum, and maximum of the pharmacokinetic parameters of esomeprazole in goats after IV and SQ administration. Table 2 displays the geometric mean, median, minimum and maximum of the pharmacokinetic parameters of esomeprazole sulfone in goats after IV and SQ administration. The bioavailability of esomeprazole after SC administration was 116%.

**Table 1:** Pharmacokinetic parameters of esomeprazole after single dose intravenous (IV: 1 mg/kg) and subcutaneous (SC: 2 mg/kg) administration in goats (*n* = 5).

**Compound (Route)**	**Parameter**	**Unit**	**Geomean**	**Median**	**Min**	**Max**
Esomeprazole	C0	μg/mL	2.324	2.876	1.015	3.609
(IV)	AUC_last_	min*ng/L	26.366	23.667	21.824	35.818
	AUC_inf_	min*ng/L	26.517	23.826	21.928	36.092
	AUMC_inf_	min*ng/L	243.632	221.706	162.258	437.975
	MRT_inf_	min	9.19	8.41	7.4	14.08
	Cl	mL/min/kg	24.9	21.9	12.6	57.2
	*T*_1/2_ (λz)	min	6.12	6.13	4.53	8.16
	λz	1/min	0.11	0.11	0.085	0.15
	*V_*z*_*	L/kg	0.23	0.19	0.12	0.67
Esomeprazole	*C*_max_	μg/mL	1.038	1.065	0.684	1.730
(SQ)	*T*_max_	min	21.69	20	20	30
	AUClast	min*ng/L	60.956	57.552	46.905	94.082
	AUC_inf_	min*ng/L	61.855	58.725	47.485	95.281
	AUMC_inf_	min*ng/L	3,196.035	3,777.278	1,951.646	4,407.353
	MRT_inf_	min	51.67	52.11	40.32	70.64
	*T*_1/2_ (λz)	min	29.16	26.81	24.02	38.72
	λz	1/min	0.024	0.026	0.018	0.029
	*V_*z*_*/F	L/kg	0.90	0.78	0.60	2.4

*C0*, calculated concentration at time zero of IV administration; *AUC*_*last*_, area under the curve calculated at the last time point; *AUC*_*inf*_, area under the curve extrapolated to infinity; *AUMC*_*inf*_, area under the moments curve extrapolated to infinity; *MRT*_*inf*_, mean residence time extrapolated to infinity; *CL*, plasma clearance; *T*_1/2_ (λ*z*), elimination half-life; *Vz*, volume of distribution; *Cmax*, maximum plasma concentration after SQ administration; *Tmax*, time to reach maximum plasma concentration after SQ administration; *Vz*/*F*, volume of distribution accounting for bioavailability.

**Table 2:** Pharmacokinetic parameters of esomeprazole sulfone after single dose intravenous (IV: 1 mg/kg) and subcutaneous (SC: 2 mg/kg) administration of esomeprazole in goats (*n* = 5).

**Compound (route)**	**Parameter**	**Unit**	**Geomean**	**Median**	**Min**	**Max**
Esomeprazole	*C*_max_	μg/mL	0.032	0.027	0.015	0.126
Sulfone	*T*_max_	min	6.6	5	5	10
(IV)	AUC_last_	min*ng/L	0.519	0.345	0.183	3.357
	AUC_inf_	min*ng/L	2.369	2.633	1.484	3.782
	MRT_inf_	min	44.65	45.87	35.36	56.37
	*T*_1/2_ (λz)	min	37.51	37.52	36.83	38.2
Esomeprazole	*C*_max_	μg/mL	0.017	0.016	0.010	0.043
Sulfone	*T*_max_	min	18.61	20	5	60
(SQ)	AUC_last_	min*ng/L	0.201	0.390	0.025	0.430

*C*_*max*_, maximum plasma concentration after SQ administration; *T*_*max*_, time to reach maximum plasma concentration after SQ administration; *AUC*_*last*_, area under the curve calculated at the last time point; *AUC*_*inf*_, area under the curve extrapolated to infinity; *MRT*_*inf*_, mean residence time extrapolated to infinity; *T*_1/2_ (λz), elimination half-life.

**Figure 1:**
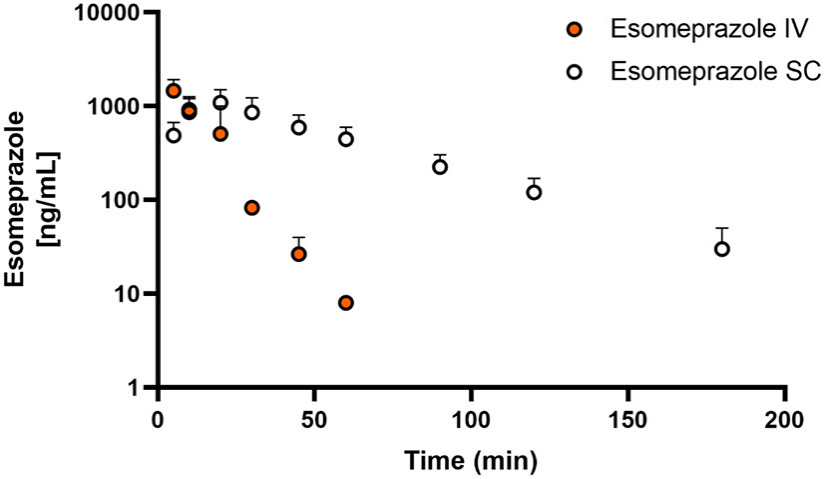
Mean plasma esomeprazole concentration (logarithmic scale) vs. time (hr) profiles for adult goats (*n* = 5) following intravenous (IV) single dose administration of 1.0 mg/kg of esomeprazole (orange) or subcutaneous (SC) single dose administration of 2.0 mg/kg of esomeprazole (white). Mean is represented by a circle with upward standard error bars.

**Figure 2:**
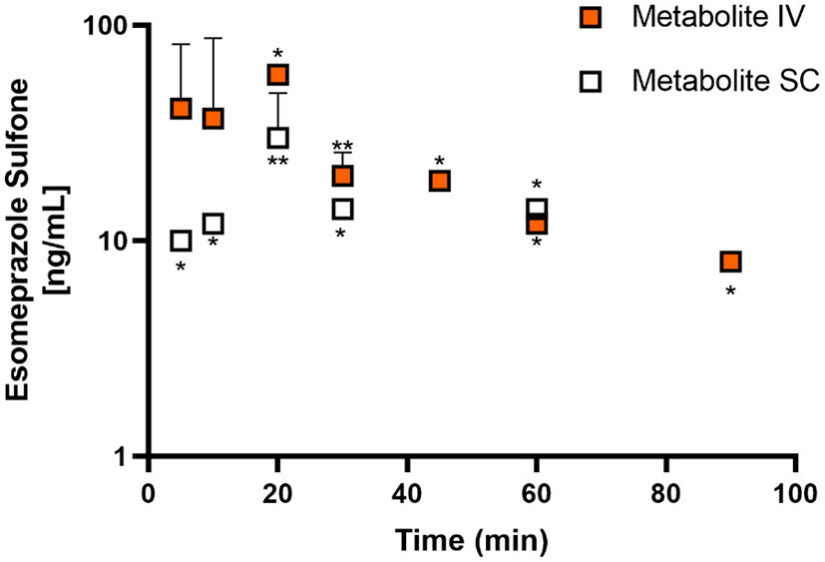
Mean plasma esomeprazole sulfone concentration (logarithmic scale) vs. time (hr) profiles for adult goats (*n* = 5) following intravenous (IV) single dose administration of 1.0 mg/kg of esomeprazole (orange) or subcutaneous (SC) single dose administration of 2.0 mg/kg of esomeprazole (white). Mean is represented by a circle with upward standard error bars. * indicates n for the time point if <5 animals (*: *n* = 1; **: *n* = 2).

Figures 1, 2 display the time vs. concentration curves for esomeprazole and esomeprazole sulfone respectively.

## Discussion

Esomeprazole is the only optical isomer of omeprazole used in veterinary medicine, and although many proton pump inhibitors are reported in clinical practice, there is not enough literature about its pharmacokinetic or pharmacodynamic properties, especially in goats, to formulate treatment plans. In this study, concentrations of esomeprazole, when administered IV and SC, were not detected after 4 h in any animal. When a single dose of esomeprazole (1 mg/kg) is administered IV it is characterized by rapid elimination in goats compared to other veterinary species, and in comparison, to other proton pump inhibitors (6). The only species with reported pharmacokinetic parameters for esomeprazole is canine, which we utilized for comparison in this study (18, 19). Although there are many equine pharmacodynamic studies, there are no published equine pharmacokinetic studies. Table 3 displays the PK parameters of esomeprazole in dogs compared to this study. The elimination half-life of IV esomeprazole in the goats of this study when administered at 1 mg/kg was 6.12 min or 0.1 h. The half-life of SC esomeprazole in the goats in our study, when administered at 2 mg/kg, was 29.16 min or 0.49 h. This is lower than canine intravenous and subcutaneous esomeprazole administration at 1 mg/kg, which was 0.73 and 0.9 h, respectively. The difference between the half live of IV vs. SC administration is most likely due to flip flop kinetics with absorption from the extravascular administration overlapping with elimination.

**Table 3:** Comparison of the PK parameters reported from the canine literature and the caprine subjects of this study.

**Compound** **(Route)**	**Parameter**	**Unit**	**Canine** **(1 mg/kg; IV) (20)**	**Canine** **(1 mg/kg; SQ) (20)**	**Goat** **(1 mg/kg; IV)**	**Goat** **(2 mg/kg; SQ)**
Esomeprazole	C0/C_max_	μg/mL	-	2.62	2.32	1.04
	AUC_last_	hr*μg/mL	3.82	4.07	0.44	1.02
	AUC_inf_	hr*μg/mL	3.82	4.07	0.44	1.03
	Cl	L/hr/kg	0.3	NA	1.50	NA
	*T*_1/2_ (λz)	Hr	0.73	0.90	0.1	0.49
	*V_*ss*_*	L/Kg	0.27	NA	0.23	NA
	*F*	%	NA	106	NA	116

*C*_*max*_, peak plasma concentration; *AUC*, area under the plasma concentration-time curve, extrapolated to last time point (AUC_last_) and to infinity (AUC_inf_); *CL*, systemic plasma clearance; *T*_1/2_ (λz), terminal elimination half-life; *V*_ss_, steady state volume of distribution; *F*, bioavailability.

This study utilized both IV and SC administration to investigate how the half-life and bioavailability are affected by route of administration. In a clinical setting, proton pump inhibitors are heavily utilized but highly variable in the amount of time they cause therapeutic effects. Due to previous pantoprazole studies on small ruminants, it was identified that SC administration had a significantly longer plasma half-life than IV administration (7). The results of this study provided a baseline for investigating multiple routes of esomeprazole administration. While IV administration is common in hospital settings, it is more technical than SC administration, and as such SC administration may be an ideal route for administration in the field by producers. An interesting finding of this study was a SC bioavailability of >100%. While this is unusual, it could be due to distribution, sampling schedule (possibly due to the later sampling time points having more time in between collection, potentially introducing artifact into area under the curve calculation), and a similar observation has been identified in dogs administered esomeprazole by an extravascular route. The clearance of esomeprazole of the goats within our study was 1,050.31 mL/min (24.95 mL/min/kg), which is consistent with its extremely rapid elimination half-life. Due to the lack of pharmacokinetic studies of esomeprazole in veterinary medicine, it is currently difficult to determine species to species variability of esomeprazole metabolism, clearance and effects. In humans, esomeprazole is thought to be metabolized in the liver by CYP2C19 enzymes, with the majority of the drug excreted in the urine (21).

In humans, patients with gastro-esophageal reflux disease are of interest for the investigation of esomeprazole. Esomeprazole has prolonged action within the body and rapid absorption in humans, along with lower inter-patient variability when compared to omeprazole. The most reported side effects of esomeprazole include headache, respiratory disease, diarrhea, abdominal pain and nausea (21). It is reported in humans that maintenance of esomeprazole therapy daily for up to 6 months was generally well-tolerated, and that a significantly increased number of people were treated for gastro-esophageal reflux disease and ulceration after 8 weeks of treatment (21). However, in some cases, proton-pump inhibitor medications, when used in veterinary hospitals, may be intended to reduce the development of ruminal ulceration rather than treat active ulceration. These findings are consistent with a retrospective investigation studying the safety of pantoprazole in hospitalized ruminant species (7) although additional prospective safety investigations are necessary to completely assess the safety profile of this drug in goats.

In a human study, there is a reported group of people that display poor metabolism of esomeprazole, resulting in decreased systemic clearance (21). This difference in metabolic capacity be similar for goats, as our study identified one animal with no detectable sulfone metabolite after SC esomeprazole administration. In humans, the sulfone metabolite is not active within the body (21). The significance of the sulfone metabolite in goats is currently unknown.

A major limitation of this investigation is the small sample size of animals, but many pharmacokinetic studies of 4–6 animals are sufficient for describing necessary pharmacokinetic parameters (22). All goats involved in the study were unrelated and while 2 goats were pygmy-crosses, the other goats were pygmies, so breeds utilized were similar. Esomeprazole is rapidly eliminated in the goat, both with intravenous and subcutaneous administration, which is a limitation to describing the elimination phase accurately. While this study had increased sampling frequency compared to other proton-pump inhibitor pharmacokinetic studies in small ruminants, the rapid elimination of esomeprazole may potentially require a different sample schedule or more sensitive methods to further characterize the elimination phase. The clinical significance of this rapid elimination is currently unknown. Because esomeprazole irreversibly binds to the proton pump, sustained levels may be unnecessary to meet treatment goals. Administering higher dosages could be investigated in further studies if pursued to potentially prolong drug exposure. Additional studies should consider a larger population, as well as population pharmacokinetic modeling (such as non-linear mixed effects) (23) to further elucidate variability amongst goats administered esomeprazole.

Further studies of the metabolism and pharmacodynamic effects of esomeprazole in goats will be needed to understand esomeprazole and the effects of its metabolites within small ruminant species. It is not currently known if there is a specific concentration of esomeprazole in circulation that needs to be reached for a certain period of time for desired effects. Future studies should investigate the efficacy of esomeprazole in goats to determine the potential for differenced in metabolic capacity of this drug within goats, as well as determine the effects of esomeprazole on the gastrointestinal tract, the ruminant gastrointestinal microbiome, and changes in abomasal acidity. The use of esomeprazole in small ruminants would be considered extra-label, which indicates that future studies involving the investigation and determination of withdrawal times would be necessary. Future studies could also investigate the effect of breed and gender on the pharmacokinetics of esomeprazole to determine what variation may be possible. Currently, it is also unknown if esomeprazole has any potential for epigenetic effects (24). Further investigation of multiple dosing of esomeprazole in small ruminants is also warranted for future studies.

In conclusion, esomeprazole administered either intravenously or subcutaneously in goats appears to be very rapidly eliminated. Although rapidly eliminated, the administration of this drug is well tolerated. Elimination half-life and plasma clearance appear to be significantly faster than reported in canines. Esomeprazole sulfone, the metabolite of esomeprazole, was detectable in plasma for <4 h regardless of the route of administration. Esomeprazole was metabolized significantly faster than pantoprazole in small ruminants (6). A larger population of animals along with tissue sampling strategies should be considered in future studies to completely describe the pharmacokinetics of esomeprazole in goats. Future studies looking at the pharmacodynamics of esomeprazole should be considered.

## Data availability statement

The original contributions presented in the study are included in the article/supplementary material, further inquiries can be directed to the corresponding author.

## Ethics statement

The animal study was reviewed and approved by Institutional Animal Care and Use Committee, University of Tennessee.

## Author contributions

RF, JS, AK, and SC developed the experimental design. RF, WS-G, RR, BF, OE, JG, JS, and LH contributed to logistical support, assessment of animal health, and data collection. SC and JB developed the analytical method for determination of concentration. JS performed the pharmacokinetic analysis. All authors contributed to manuscript construction. All authors contributed to the article and approved the submitted version.
